# Activated STAT3 Could Reduce Survival in Patients with Esophageal Squamous Cell Carcinoma by Up-regulating VEGF and Cyclin D1 Expression

**DOI:** 10.7150/jca.38798

**Published:** 2020-01-20

**Authors:** Nan Zhang, Min Zhang, Zhou Wang, Wei Gao, Zhi-Gang Sun

**Affiliations:** 1 Department of Oncology, Jinan Central Hospital Affiliated to Shandong University; Jinan Central Hospital Affiliated to Shandong First Medical University; Jinan 250013, P.R. China.; 2 Department of Dermatology, Jinan Central Hospital Affiliated to Shandong University; Jinan Central Hospital Affiliated to Shandong First Medical University; Jinan 250013, P.R. China.; 3 Department of Thoracic Surgery, Provincial Hospital Affiliated to Shandong University, Jinan 250021, P.R. China.; 4 Department of Pathology, Jinan Central Hospital Affiliated to Shandong University; Jinan Central Hospital Affiliated to Shandong First Medical University; Jinan 250013, P.R. China.; 5 Department of Thoracic Surgery, Jinan Central Hospital Affiliated to Shandong University; Jinan Central Hospital Affiliated to Shandong First Medical University; Jinan 250013, P.R. China.

**Keywords:** STAT3, p-STAT3, Esophageal squamous cell cancer, Western Blot, Immunohistochemistry.

## Abstract

Signal transduction and activators of transcription factor (STAT) 3 is associated with a poor prognosis in certain types of cancer. The purpose of the present study was to investigate the clinical and prognostic significance of STAT3/p-STAT3 expression in esophageal squamous cell cancer (ESCC) patients. A total of 71 patients were enrolled in the study. STAT3 and p-STAT3 expression were detected by Western Blot and immunohistochemistry assays. Stattic, the STAT3 inhibitor, was used to block the activation of STAT3 in ESCC cell lines Eca-109 and Kyse-30, and the CCK8 assay was performed to detect the effect of Stattic on the viability of ESCC cells. The expression of associated genes was assessed by RT-PCR and Western blot at RNA and protein levels, respectively. STAT3 expression was correlated with infiltration degree (pT) and pTNM. And p-STAT3 expression was correlated with pT, lymphatic metastasis (pN) and pTNM. The expression of VEGF, Bcl-xl and Cyclin D1 was up-regulated in ESCC tissues and positively correlated with p-STAT3 level, besides Bcl-xl. *In vitro*, Stattic inhibited the viability of Eca-109 and Kyse-30 cells in a dose- and time- dependent manner, and significantly inhibited the expression of VEGF, Bcl-xl and CyclinD1 at mRNA and protein level. The 5-year survival rate of the 71 patients was significantly associated with pT, pN, pTNM stage, p-STAT3 level, VEGF expression and Cyclin D1 expression. pN and p-STAT3 expression were independent relevant factors. Our results showed that p-STAT3 might serve as an essential biomarker for tumor invasion and metastasis in ESCC.

## Introduction

Esophageal cancer is one of the ten malignant tumors in China, of which esophageal squamous cell carcinoma (ESCC) is the major histological type. Esophagostomy is the major treatment method at present. However, the prognosis is not satisfactory, and the 5-year survival rate of the ESCC patients is less than 30% 1. The tumor-node-metastasis (TNM) staging system, according to histopathologic findings, lacks sufficient predictive value as significant differences in survival are often observed for the same TNM stage. Therefore, a combination of a number of biomarkers to more accurately distinguish patients with ESCC with poor survival would be valuable 2, 3.

Signal transduction and activators of transcription factor (STAT) 3, a member of the STAT family, is frequently regarded as an oncogene 4-6. STAT3 can be activated via phosphorylation events within the Janus kinase-STAT, producing tyrosine or serine phosphorylated STAT3 (p-STAT3) 7, 8. p-STAT3 is able to increase the expression levels of certain target genes, including vascular endothelial growth factor (VEGF), Cyclin D1 and Bcl-xL, etc 9-12. STAT3 constitutively activation is frequently observed in cancers, including lung cancer, liver cancer, gastric cancer and renal cell carcinoma 13. Increasing number of studies show that STAT3 may serve as one of the oncogenic critical factors and is associated with a poor prognosis in certain types of cancer. For instance, it has been reported that elevated STAT3 expression is associated with poor prognosis in gastric cancer, lung cancer and live cancer 13. Additionally, STAT3 has been revealed to be crucial involved in cancer cell proliferation, invasion, migration and apoptosis 14, 15.

A limited number of studies have previously demonstrated the clinical features of STAT3/ p-STAT3 in ESCC. Therefore, the present study investigated the association between STAT3 expression, p-STAT3 level and the clinical features of ESCC patients. Furthermore, the present study also investigated the potential prognostic value of STAT3/p-STAT3 expression for predicting the survival of ESCC patients.

## Material and Methods

### Patients and samples

We obtained ESCC specimens from the 71 patients who were enrolled in this study at the department of thoracic surgery East Ward, Provincial Hospital Affiliated to Shandong University from September 2008 to January 2009. The inclusion criteria were as follows: 1) patients underwent complete resection and postsurgical pathology proved ESCC; 2) patients were diagnosed as postoperative pathologic stage I-III; 3) patients accepted no preoperative radiotherapy or chemotherapy; 4) patients had no serious surgical contraindication. The clinicopathalogical characteristics of the patients are listed in Table 1. This study was approved by Shandong University Ethics Committee. Adjacent non-tumorous esophageal tissues were used as the control tissues. Each specimen was divided into two parts. At least 0.5cm×0.5cm×0.5 cm ESCC specimens were used for western blot assay. The other ESCC specimen was used for histopathologic examination and immunohistochemistry.

### Cell culture

The human ESCC cell lines Eca-109 and Kyse-30 were obtained from the Cell Bank of Chinese Academy of Sciences (Shanghai, China). Cells were cultured in Dulbecco's Modified Eagle's Medium (DMEM; HyClone, Thermo Fisher Scientific, Waltham, MA, USA) contained 10% fetal bovine serum (FBS; Gibco, Thermo Fisher Scientific) and antibiotics (100U/mL, Sigma‑Aldrich, Germany) at 37°C with 5% CO_2_.

### Immunohistochemistry

Immunohistochemistry staining for STAT3, p-STAT3, VEGF, CyclinD1, and Bcl-xl protein were detected by streptavidin-peroxidase method (SP method). Being fixed in 10% neutral buffered formalin, the tissue specimens were processed routinely. Immunohistochemistry analysis was performed using rabbit antibody against human STAT3 (Spring Bioscience, USA), rabbit antibody against human p-STAT3 (tyr705) (Santa Cruz Biotechnology, USA), rabbit antibody against human VEGF (Zhongshanjinqiao Biotechnology, China), rabbit antibody against human Cyclin D1 (Zhongshanjinqiao Biotechnology), rabbit antibody against human Bcl-xL (Bioss Biotechnology, China), and visualized by the Envision System (Dako). STAT3, p-STAT3, VEGF, CyclinD1 and Bcl-xL were scored with immunohistochemistry using system as previously described 16, 17. Briefly, a score of 3 indicated that >50% of the cells exhibited mild to moderate staining intensity, or >20% of cells exhibited strong staining intensity; a score of 2 indicated that 20‑50% of cells demonstrated mild to moderate staining intensity, or 20% of cells exhibited strong staining intensity; a score of 1 indicated that <20% of cells demonstrated mild to moderate staining intensity; and a score of 0 indicated that no staining was present. A score of ≥2 demonstrated positive expression.

### Western Blot

The micrograms of the proteins from each sample or Eca-109 and Kyse-30 cells treated with Stattic for 24h were separated by SDS-PAGE and transferred to PVDF membranes (Millipore, France). The membranes were blocked with 5 % skim milk and incubated with primary antibodies against STAT3 (Spring Bioscience, USA), p-STAT3 (S727; Santa Cruz, USA), VEGF (Proteintech Group, IL, USA), CyclinD1 (Proteintech Group), Bcl-xL (Proteintech Group), and β-actin (Bioss Biotechnology) at 4 °C overnight, following by incubation with HRP-conjugated secondary antibodies (Santa Cruz, USA) for 1 h at room temperature. Immunoblotted proteins were visualized by ECL reagents, and the signals were detected by Alphamager 2200 imaging system (Alphamager, USA) and Image J analysis software.

### Follow-up

40 patients received postoperative chemotherapy, and 24 patients received postoperative radiotherapy. We examined the patients 3 to 4 months during the first 3 years and half a year thereafter. The patients only died of cancer were enrolled in the prognostic analysis.

### CCK8 assay

For dose-dependent assay, cells were seeded into 96-well plates and treated with different concentrations of Stattic (0, 0.05, 0.1, 0.2, 0.3, 0.4, 0.5, 0.6, 0.8, 0.9, and 1 µM, Med Chem Express, USA) for 24h. After treatment, 10 μl of Cell Counting Kit-8 reagent (CCK8; Beijing Solarbio Science & Technology, Beijing, China) were added into each well, following by incubation at 37°C for 90 min. The optical density (OD) was measured at 450nm. Following treatment with Stattic of 24h, cell viability was measured at 0, 24, 48, and 72 h, respectively.

### Real-time polymerase chain reaction (RT-PCR)

After being treated for 24h, total RNA from Eca-109 and Kyse-30 cells was extracted using Ultrapure RNA Kit (CWBIO, Beijing, China) and reverse transcribed in complementary cDNA using the HiFiScript cDNA Synthesis Kit (CWBIO) according to the manufacturer's instructions. RT products were then used as templates to examine the expression of target gene mRNA using real-time PCR with the SYBR Premix Ex Taq II kit (Takara, Shiga, Japan). The primer sequences were as follows: VEGF, 5'- GCTACCTCAGCAAGACGTTATT -3' (forward), 5'- ATCGGCAGGAAGTGTGATTG -3' (reverse); CyclinD1, 5'-GTGCCACAGATGTGAAGT -3' (forward), 5'- GTAGGACAGGAAGTTGTTGG-3' (reverse); Bcl-xL, 5'- GGTGGTTGACTTTCTCTCCTAC -3' (forward), 5'- TCTCCGATTCAGTCCCTTCT -3' (reverse); β-actin, 5'- CCCGAGCCGTGTTTCCT -3' (forward), 5'- GTCCCAGTTGGTGACGATGC -3' (reverse). β-actin was used as internal gene. The obtain data was analyzed according to the sample threshold cycle (Ct) value from three independent experiments.

### Statistical analysis

Enumeration data were analyzed using χ^2^ test or Fisher's exact probability test. Measurement data was represented with Mean ± standard deviation (SD) and t-rest. Univariate analysis was performed by modeling Kaplan-Meier survival curves. The log-rank test was used to calculate the survival rate. Multivariate analysis was carried out by the use of the Cox proportional hazard model. The difference between two groups were estimated with student's *t* test. All statistical data were analyzed using SPSS (version 13; SPSS, Inc., Chicago, IL, USA). P<0.05 was considered to indicate a statistically significant difference.

## Results

### Correlation between STAT3/ p-STAT3 expression and clinical features of ESCC

**Figure 1:**
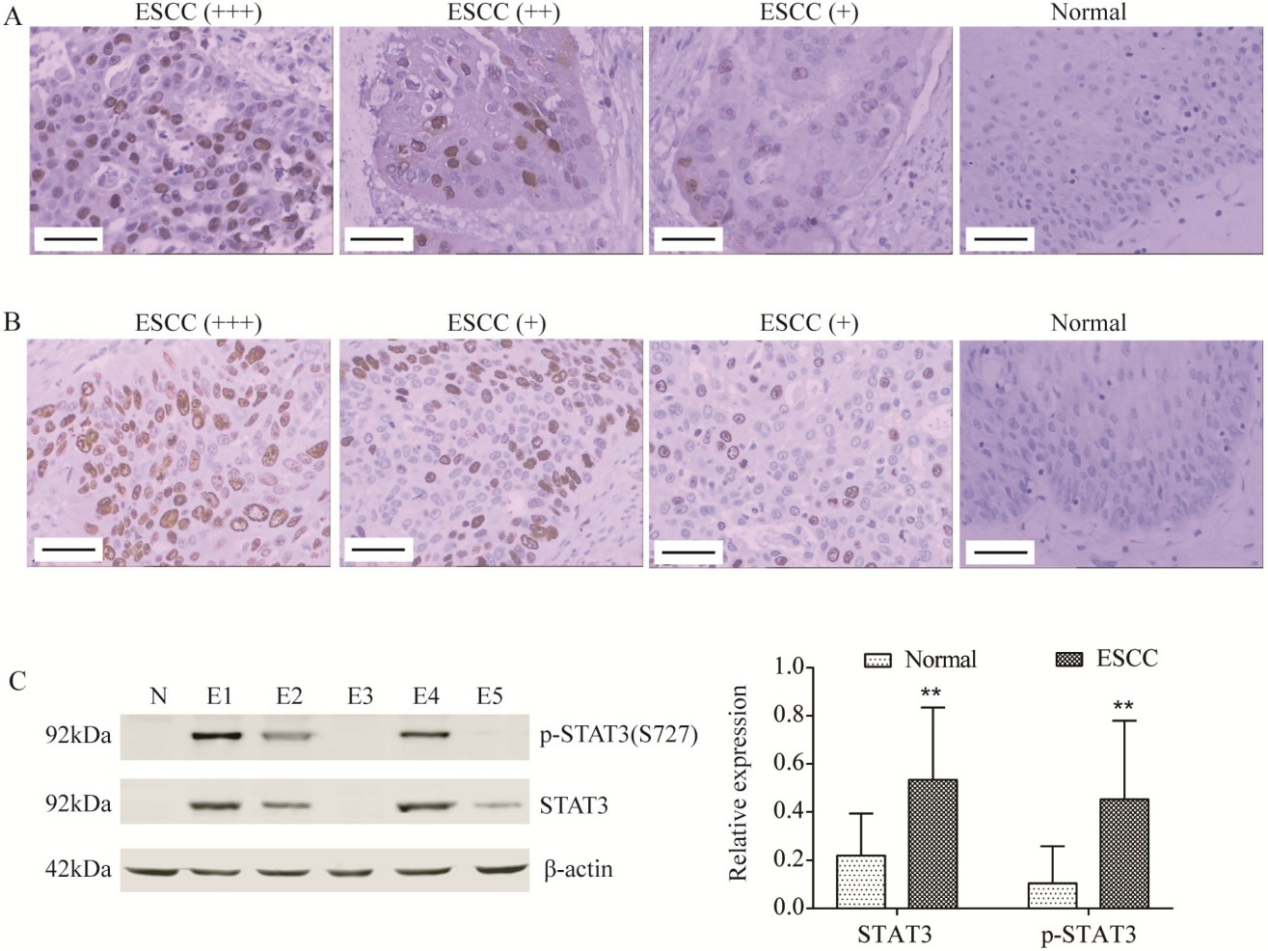
** STAT3 and p-STAT3 was up-regulated in ESCC tissues.** (A, B) STAT3 (A) and p-STAT3 (B) expression was determined by immunohistochemistry (Magnification 200). From left to right, ESCC tissues with strongly positive expression (+++), positive expression (++), weak positive expression (+) and normal tissue (control). Scale Bar=100 µm. (C) STAT3 and p-STAT3 expression was determined by Western blot. N, normal tissue; E1, pT3 ESCC tissue; E2, pT2 ESCC tissue; E3, pT1 ESCC tissue; E4, ESCC tissue with lymphatic metastasis; E5, ESCC tissue without lymphatic metastasis. ^**^P<0.01 compared to normal.

As indicated in Figure 1A, the positive signal of STAT3 protein was located in cytoplasm and nucleus. The expression of STAT3 was correlated with infiltration degree (pT; pT1 25.0% *vs*. pT2 84.8% *vs*. pT3 90.5%; *P*<0.01, Table 1) and pTNM stage (pI, 25.0% *vs*. pII, 87.3% *vs*. pIII, 83.3%; *P*<0.01, Table 1). The positive signal of p-STAT3 protein, the active form of STAT3, was located in cell nucleus (Figure 1B). The level of p-STAT3 was correlated with pT (pT1 25.0% *vs*. pT2 52.2% *vs*. pT3 90.5%; *P*<0.05, Table 1), lymphatic metastasis (pN; pN- 48.9% *vs*. pN+84.6%; *P*<0.01, Table 1) and pTNM stage (pI, 25.0% *vs*. pII, 40.0% *vs*. pIII, 91.7%; *P*<0.01, Table 1). There was a positive correlation between STAT3 expression and p-STAT3 in the cancerous tissue group (r=0.421, *P*<0.01, Table 4).

The expression of STAT3 and level of p-STAT3 in ESCC tissues were also detected using Western blot (Figure 1C). Moreover, the expression of STAT3 was correlated with pT and pTNM stage (both *P*<0.01, Table 2), and the level of p-STAT3 was correlated with pT, pN and pTNM stage (both *P*<0.01, Table 2), which was consistent with immunohistochemical results.

### Correlation between STAT3/p-STAT3 expression and downstream proteins in ESCC

**Figure 2:**
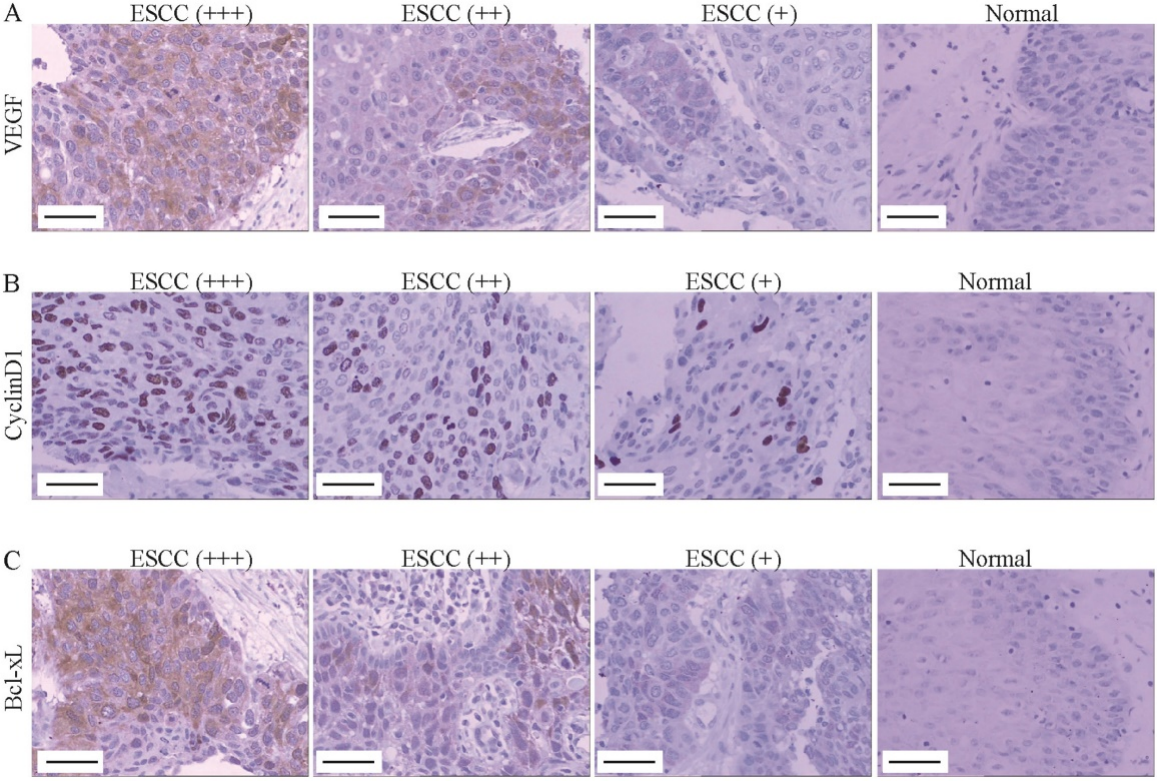
** VEGF, Cyclin D1 and Bcl-xL was up-regulated in ESCC tissues.** (A-C) The VEGF (A), Cyclin D1 (B) and Bcl-xl (C) expression was determined by immunohistochemistry (Magnification 200). From left to right, ESCC tissues with strongly positive expression (+++), positive expression (++), weak positive expression (+) and normal tissue (control). Scale Bar=100 µm.

Furthermore, the expression of downstream proteins of STAT3 was also assessed using immunohistochemistry assay. As indicated in Figure 2A, the positive signal of VEGF protein was located in cytoplasm, which was significantly correlated with pT (pT1 25.0% *vs*. pT2 56.5% *vs*. pT3 85.7%; *P*<0.05, Table 3), lymphatic metastasis (pN; pN- 48.9% *vs*. pN+ 88.5%; *P*<0.01, Table 3) and pTNM stage (pI, 25.0% *vs*. pII, 51.1% *vs*. pIII, 91.7%; *P*<0.05, Table 3). The positive signal of Cyclin Dl protein was located in cell nucleus and up-regulated in ESCC tissues, which was significantly correlated with lymphatic metastasis (pN; pN- 44.4% *vs*. pN+76.9%; *P*<0.05, Figure 2B, Table 3). The positive signal of Bcl-xL protein was located in cytoplasm, which was significantly correlated with pT (pT1 25.0% *vs*. pT2 58.7% *vs*. pT3 90.5%; *P*<0.05, Figure 2C, Table 3). Importantly, we found that there was a positive correlation between STAT3 protein expression and VEGF protein expression (r=0.255, *P*<0.0, Table 4), but the expression of STAT3 was not correlated with the expression of cyclinD1 and Bcl-xL (both *P*>0.05, Table 4). Moreover, we found that the level of p-STAT3 was positively correlated with the expression of VEGF protein (r=0.352, *P*<0.01, Table 4) and CyclinD1 protein (r=0.305, *P* <0.05, Table 4) in the cancerous tissue group, except for Bcl-xL (*P*>0.05, Table 4).

### Inhibition of STAT3 activation inhibited downstream proteins expression in ESCC cells *in vitro*

**Figure 3:**
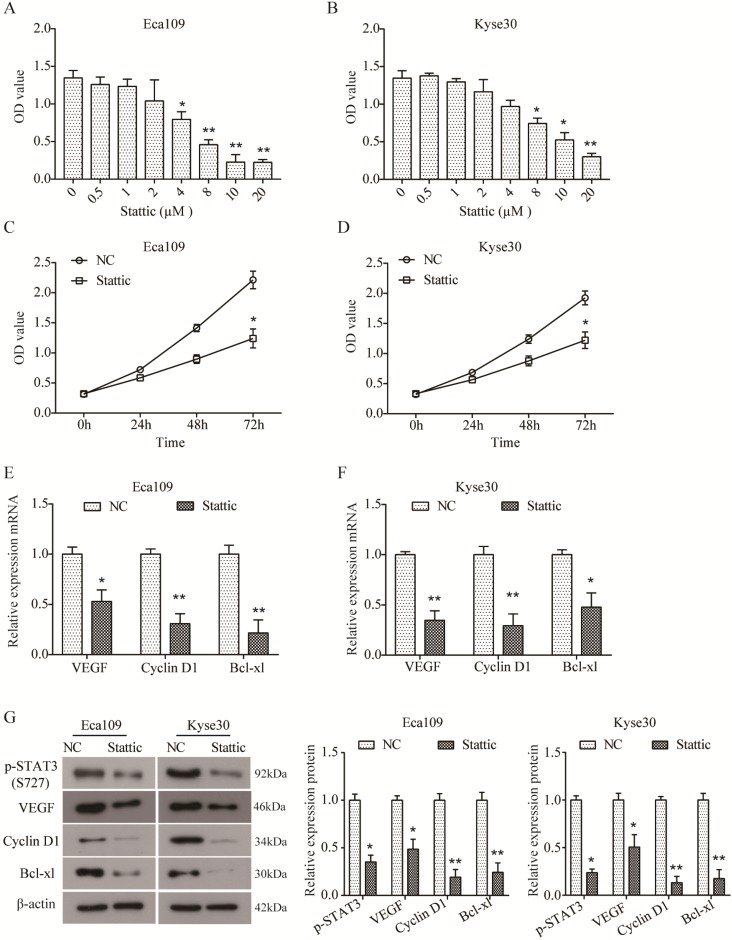
** Stattic inhibited cell viability and expression of VEGF, Cyclin D1 and Bcl-xl.** (A, B) Eca109 (A) and Kyse30 (B) cells were treated with different concentrations of Stattic for 24 h, and CCK8 assay was performed to assess cell viability. (C, D) Following treatment with Stattic for 0, 24, 48 and 72 h, the viability of Eca109 (C) and Kyse30 (D) cells was examined using CCK8 assay. (E, F) After being treated with Stattic for 24 h, the expression of VEGF, Cyclin D1 and Bcl-xL mRNA was detected using RT-PCR in Eca109 (E) and Kyse30 (F) cells. (G) The expression of p-STAT3, VEGF, Cyclin D1 and Bcl-xl protein was detected using Western blot in Eca109 and Kyse30 cells. NC, cells treated with DMSO; Stattic, cells treated with Stattic. All data from three independent experiments was quantified. ^*^P<0.05, ^**^P<0.01 compared to NC.

In order to further investigate the role of STAT3 in ESCC, two ESCC cell lines Eca109 and Kyse30 were treated with different concentrations of Stattic (0, 0.5, 1, 2, 4, 8, 10, and 20 µM) to inhibit the activation of STAT3. As indicated by CCK8 assay, Stattic inhibited the viability of Eca109 (Figure 3A) and Kyse30 (Figure 3B) cells in a dose-dependent manner. And the IC50 of Stattic was 5.532 μM for Eca109 cells and 8.785 μM for Kyse30 cells. As a result, 3 μM of Stattic was used for the subsequent experiments for the appropriate effect on Eca109 cells and 5 μM of Stattic for Kyse30 cells, DMSO was used as the negative control (NC). Moreover, as shown in Figure 3C and D, Stattic also inhibited the viability of Eca109 and Kyse30 cells in a time-dependent manner. Our data demonstrated that compared to the NC group, the mRNA expression of VEGF, Cyclin D1 and Bcl-xl was significantly down-regulated in Stattic-treated Eca109 cells (both *P*<0.05, Figure 3E). Similar results were also observed in Kyse30 cells (Figure 3F). In addition, Western blot results further suggested that Stattic could obviously reduce the level of p-STAT3 and inhibit the expression of VEGF, Cyclin D1 and Bcl-xl in Eca109 and Kyse30 cells at protein level (both *P*<0.05, Figure 3G). Overall, these data indicated that blocking the activation of STAT3 can inhibit the growth of ESCC through down-regulation of VEGF, Cyclin D1 and Bcl-xl.

### Correlation between STAT3/ p-STAT3 expression and prognosis of ESCC

**Figure 4:**
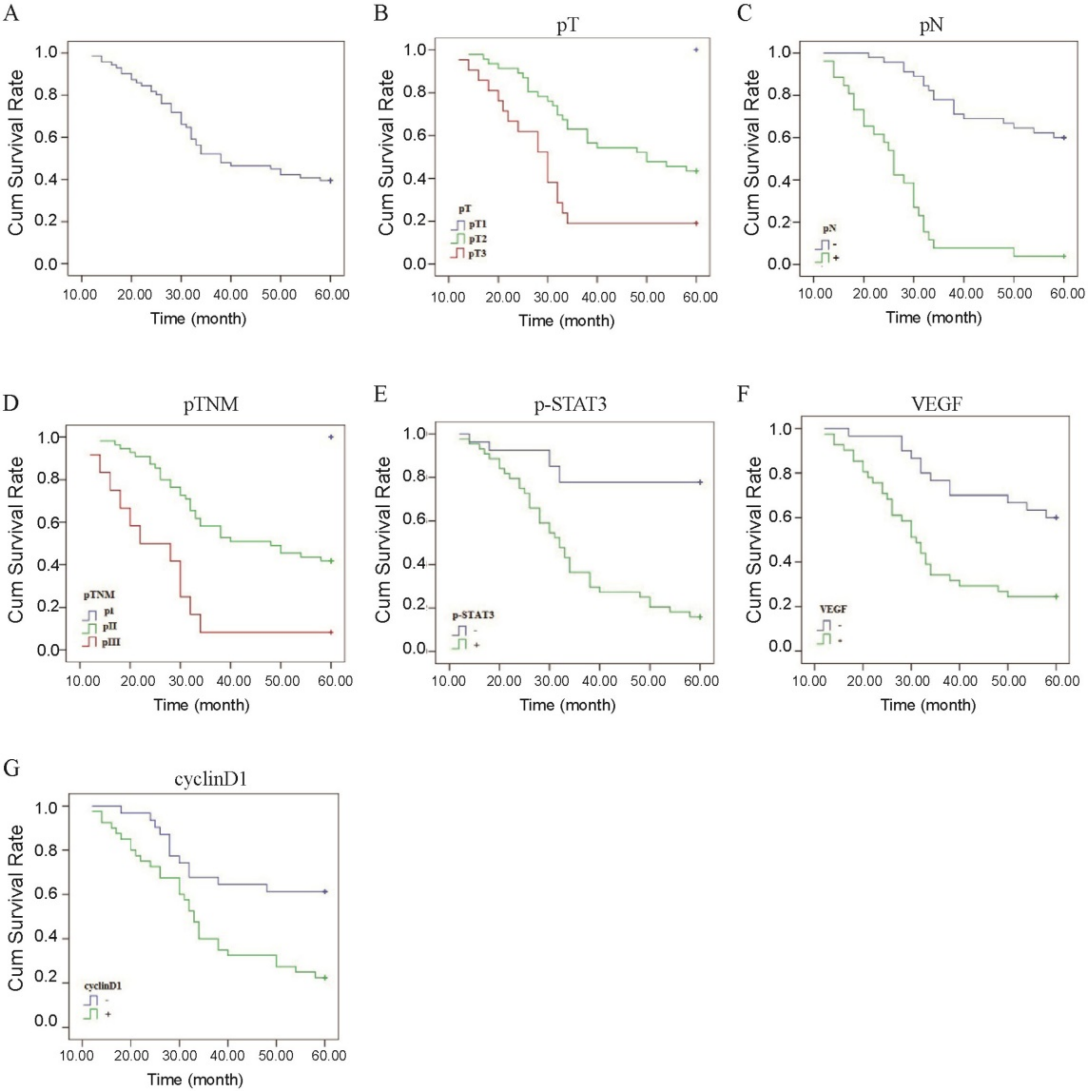
** Correlation between p-STAT3/VEGF/cyclinD1 expression and prognosis of ESCC.** (A) The Kaplan-Meier survival curve of 71 cases of patients with ESCC. (B) Survival curves of ESCC patients with different pT. Blue, patients with pT1; green, patients with pT2; red, patients with pT3. (C) Survival curves of ESCC patients with negative or positive pN. Blue, patients with negative pN; green, patients with positive pN. (D) Survival curves of ESCC patients with different pTNM. Blue, patients with pI; green, patients with pII; red, patients with pIII. (E) Survival curves of ESCC patients with positive and negative expression of p-STAT3 protein. Blue, p-STAT3 negative expression; green, p-STAT3 positive expression. (F) Survival curves of ESCC patients with positive and negative expression of VEGF protein. Blue, VEGF negative expression; green, VEGF positive expression. (G) Survival curves of ESCC patients with positive and negative expression of Cyclin D1 protein. Blue, Cyclin D1 negative expression; green, Cyclin D1 positive expression.

The Kaplan-Meier method indicated that the 5-year survival rates of the 71 patients was 39.4% (Figure 4A). In univariate analysis by the log-rank test, the 5-year survival rate in patients after operation was significantly associated with pT (*P*<0.01, Figure 4B, Table 5), pN (*P*<0.01, Figure 4C, Table 5), pTNM stage (*P*<0.01, Figure 4D, Table 5), level of p-STAT3 (*P*<0.01, Figure 4E, Table 5), VEGF expression (*P*<0.01, Figure 4F, Table 5), and CyclinD1 expression (*P*<0.01, Figure 4G, Table 5). However, there were no statistically significant correlations with gender, age, tumor length; tumor location, histological differentiation, STAT3 expression, Bcl-xl expression, radiotherapy, and chemotherapy were demonstrated for the 5-year survival rate (Table 5). The results of Cox regression multivariate analysis confirmed that pN and level of p-STAT3 were independent relevant factors (Table 6).

## Discussion

STAT3 is regarded as a primary mediator of tumorigenesis and serves an important function in the proliferation, apoptosis and hyperplasia of tumor cell 14, 15. Constitutively activated STAT3 has been identified in certain types of cancer, including ESCC. *In vitro*, Wang et al. demonstrated that the STAT3 signaling pathway was constitutively activated in ESCC cells, and the expression of STAT3, VEGF and Bcl-2 were overexpressed in ESCC cell lines 18. S Timme et al. reported that STAT3-regulated genes (STAT3, p-STAT3) were involved in ESCC cell proliferation and migration 19. Activate STAT3 might affect STAT3 target genes expression and promote the growth of ESCC cells, which could be blocked by STAT3 inhibitor and specific siRNA 20, 21.

However, few studies reported the clinical and prognostic significance of STAT3 and p-STAT3 in ESCC patients. In Li's study, STAT3 protein expression was correlated with pTNM stage in ESCC patients, and dual high expression of STAT3 and Cyclin D1 predict worse survival outcome 11. You et al. reported that p-STAT3 expression was correlated with lymph node metastasis, pTNM stage and metastatic status 22. Huang et al. identified that p-STAT3 expression was significantly associated with poor prognosis in advanced esophageal cancer patients 23. In the present study, a total of 71 ESCC patients enrolled the study, the STAT3 and p-STAT3 expression were detected by immunohistochemistry and Western blot at protein level. STAT3 was located in cytoplasm and nucleus while the p-STAT3 was exclusively observed in nucleus of ESCC cells. Moreover, our data demonstrated that the expression of STAT3 and p-STAT3 was both up-regulated in ESCC tissues, and the STAT3 expression was correlated with pT and pTNM stage, the p-STAT3 level was correlated with pT, pN and pTNM stage. Xuan X et al. also reported that STAT3 and p-STAT3 were up-regulated in ESCC tissues, which was consistent with our results 24. The findings described above indicated that STAT3 activation might increase the metastasis of ESCC.

STAT3 has been revealed to regulate the expression of many target genes in tumorigenesis and progression, including VEGF, Cyclin D1 and Bcl-xL 10, 24-26. VEGF has been well known to play a crucial pro-oncogenic role in angiogenesis and tumor progression 27. Xu Q et al. reported that STAT3 is a key regulator of VEGF, targeting STAT3 can decrease the expression of VEGF 24. Cyclin D1, an essential regulator of G1/S phase of cell cycle involved in cell proliferation, is frequently up-regulated and amplified in tumors. It is revealed that STAT3 can regulate the expression of Cyclin D1 through directly targeting its promoter in colorectal cancer cells 28. Bcl-xL is a member of Bcl-2 family and functions as an anti-apoptotic protein. Bcl-xL is reported to be positively expressed in 46.8% of ESCC tissues 29. In endometrioid adenocarcinomas, Wincewicz A et al. found that the expression of STAT3 is correlated with Bcl-xL 30. In the current study, the expression of VEGF, Cyclin D1 and Bcl-xL was also assessed by immunohistochemistry. We observed that the expression of VEGF, Cyclin D1 and Bcl-xl was all up-regulated in ESCC tissues. Importantly, the level of p-STAT3 was all positively correlated to VEGF and Cyclin D1, except for Bcl-xL. Moreover, STAT3 expression was positively correlated to VEGF, but not with Cyclin D1 and Bcl-xL, which was not consistent with previous results in other cancers 28, 30. Furthermore, blocking the activation of STAT3 by Stattic, a STAT3 inhibitor, could inhibit the viability of ESCC cells and down-regulate the expression of VEGF and Cyclin D1. Additionally, we observed that the expression of Bcl-xL was also down-regulated by Stattic in Eca109 and Kyse30 cells, but its expression in tissues was not related to the expression of p-STAT3 (Table 4). The inconsistent results may be due to differences between tissues and cells, which will be further investigated in future study. Collectively, these results suggested that the activated STAT3 may promote the metastasis of ESCC by promoting the expression of VEGF and Cyclin D1.

It has been reported that STAT3 and p-STAT3 expression is associated with worse overall survival of many malignancies, including lung cancer, gastric cancer and hepatic cancer, but better prognosis of breast cancer 31. Aleskandarany M A et al. also demonstrated that the nuclear p-STAT3 overexpression is positively associated with better prognosis of breast cancer 32. In our study, to eliminate the impact of mixed factors on statistical analysis, we used both univariate and multivariate analysis to determine prognostic factors in order to make the results more objective. In this study, we observed that the 5-year survival rate of ESCC patients was 39.4 %, which was significantly associated with p-STAT3 expression, VEGF expression, Cyclin D1 expression, pT, pN, and pTNM stage. Additionally, pN and p-STAT3 expression were relevant independent factors for a poor prognosis, which was consistent with the previous study that demonstrated that p-STAT3 expression was an independent prognostic factor for progression-free-survival in ESCC 23. Taken together, these findings indicated that activation of STAT3 could serve as a biomarker of poor prognosis in ESCC.

However, the present study had still several limitations. First, in China the indications for treatment not only depend on doctors' preferences but also on patients' willingness and economic status. These factors may have influenced the relatively poor survival result observed. In the study, 26 patients received postoperative chemotherapy, 10 patients received postoperative radiotherapy, and 14 patients received combined chemoradiotherapy. However, no statistically significant correlations with postoperative chemotherapy and radiotherapy were demonstrated for the 5-year survival rate both in univariate and multivariate analysis. Secondly, the study sample was relatively small. Lastly, all the patients' histologic type was squamous cell carcinoma in the study. Because ESCC is one of the most common malignant diseases in China, meaning that the patients enrolled in the study might not be representative of the population in the world.

In sum, STAT3 expression was correlated with pT and pTNM stage. The level of p-STAT3 was correlated with pT, lymph node metastasis and pTNM stage. pN and p-STAT3 expression were relevant independent factors for the 5-year survival rate of ESCC patients. Activated STAT3 may promote the metastasis of ESCC by promoting the expression of VEGF and Cyclin D1. Collectively, these results suggest that p-STAT3 might serve as essential biomarker for tumor invasion and metastasis in ESCC.

## Figures and Tables

**Table 1:** Correlation between STAT3/p-STAT3 expression and clinical characteristics of the ESCC (immunohistochemistry).

Clinical characteristics	Patients	STAT3		*P*	p-STAT3		*P*
		(-)	(+)		(-)	(+)	
Gender				^ *^0.356			^ *^0.489
Male	61	9	52		22	39	
Female	10	3	7		5	5	
Age, years				^*^1.000			1.000
≥60	38	6	32		14	23	
<60	33	6	27		13	21	
Tumor length				^*^>0.05			^*^>0.05
<3cm	7	2	5		4	3	
3-5cm	48	6	42		16	32	
>5cm	16	4	12		7	9	
Tumor location				^*^1.000			^*^0.461
Middle	42	7	36		18	25	
Lower	29	5	23		9	19	
Differentiation				^*^>0.05			^*^>0.05
Well	12	4	8		5	7	
Moderately	45	7	38		17	28	
Poorly	14	1	13		5	9	
pT				^*^<0.01			^*^<0.05
pT1	4	3	1		3	1	
pT2	46	7	39		22	24	
pT3	21	2	19		2	19	
pN				^*^1.000			^*^0.005
_	45	8	37		23	22	
+	26	4	22		4	22	
pTNM				^*^<0.01			^*^<0.05
pI	4	3	1		3	1	
pII	55	7	48		23	22	
pIII	12	2	10		1	11	

*P*: χ^2^ test; ^*^Fisher's exact probability test.

**Table 2:** Correlation between STAT3/p-STAT3 expression and clinical characteristics of the ESCC (Western blot).

Clinical characteristics	STAT3			p-STAT3		
	Protein Assay	t, t'	*P*	Protein Assay	t, t'	*P*
Gender		0.607	0.546		1.074	0.287
Male	0.5426±0.2997			0.4697±0.3270		
Female	0.4800±0.3190			0.3500±0.3240		
Age, years		0.352	0.726		0.358	0.721
≥60	0.5459±0.3033			0.4662±0.3476		
<60	0.5206±0.3024			0.4382±0.3705		
Tumor length		0.958	0.389		0.178	0.675
<3 cm 3-5 cm >5 cm	0.3857± 0.2854 0.5458±0.3039 0.5625±0.2986			0.3286± 0.3450 0.4802±0.3137 0.4250±0.3642		
Tumor location		-0.848	0.399		-0.422	0.675
Middle	0.5093±0.3146			0.4395±0.3303		
Lower	0.5714±0.2800			0.4732±0.3267		
Differentiation		1.273	0.287		0.653	0.524
Well	0.4417 ± 0.3825			0.3583± 0.3895		
Moderately	0.5289 ± 0.2677			0.4800 ±0.2989		
Poorly	0.6286 ± 0.3221			0.4464±0.3661		
pT		17.683	0.000		7.764	0.001
pT1	0.1000±0.2000			0.0750±0.1500		
pT2	0.4630±0.2322			0.4022±0.3213		
pT3	0.7714±0.2849			0.6357±0.2860		
pN		-1.937	0.057		-3.757	0.000
-	0.4822±0.2682			0.3511±0.3335		
+	0.6231±0.3374			0.6288±0.2300		
pTNM		17.761	0.000		5.686	0.005
pI	0.1000±0.2000			0.075±0.1500		
pII	0.4927±0.2387			0.4364±0.3123		
pIII	0.8667±0.2964			0.6542±0.3130		

*P*: t or t' test.

**Table 4:** Correlation among STAT3/p-STAT3, VEGF, Cyclin D1 and Bcl-xl expression in ESCC (by immunohistochemistry).

	r_s_	*P*
STAT3 *vs*. p-STAT3	0.421	0.000
STAT3 *vs*. VEGF	0.255	0.032
STAT3 *vs*. Cyclin D1	0.206	0.085
STAT3 *vs*. Bcl-xl	0.140	0.245
p-STAT3 *vs*. VEGF	0.352	0.003
p-STAT3 *vs*. Cyclin D1	0.305	0.010
p-STAT3 *vs*. Bcl-xl	0.151	0.207
VEGF* vs*. Cyclin D1	0.193	0.107
VEGF* vs*. Bcl-xl	0.047	0.695
Cyclin D1 *vs*. Bcl-xl	0.054	0.652

**Table 3:** Correlation between VEGF, cyclinD1, Bcl-xL expression and clinical characteristics of the ESCC patients (Immunohistochemistry).

Clinical characteristics	VEGF		*P*	CyclinD1		*P*	Bcl-xL		*P*
	(-)	(+)		(-)	(+)		(-)	(+)	
Gender			^ *^0.298			^*^1.000			0.733
Male	23	38		27	34		21	40	
Female	6	4		4	6		4	6	
Age, years			1.000			1.000			0.146
≥60	15	22		14	23		13	24	
<60	14	20		17	17		12	22	
Tumor length			^*^>0.05			^*^>0.05			^*^>0.05
<3cm	2	5		1	6		3	4	
3-5cm	21	27		22	26		15	33	
>5cm	6	10		8	8		7	9	
Tumor location			0.809			0.808			0.316
Middle	17	26		18	25		13	30	
Lower	12	16		13	15		12	26	
Differentiation			^*^>0.05			^*^>0.05			^*^>0.05
Well	5	7		3	9		5	7	
Moderately	18	27		21	24		16	29	
Poorly	3	11		7	7		4	10	
pT			^*^<0.05			^*^>0.05			^*^<0.05
pT1	3	1		3	1		3	1	
pT2	20	26		20	26		19	27	
pT3	3	18		8	13		2	19	
pN			^*^0.001			^*^0.012			^*^0.613
0.613_	23	22		25	20		17	28	
+	3	28		6	20		8	18	
pTNM			^*^<0.05			^*^>0.05			^*^>0.05
pI	3	1		3	1		3	1	
pII	22	23		25	30		19	36	
pIII	1	11		3	9		3	9	

*P*, χ2 test, ^*^Fisher's exact probability test.

**Table 5:** Univariate analysis with respect to 5-year survival.

Clinical characteristics	Patients (n=71)	5-year survival (%)	*P*
Gender			0.169
Male	61	36.1 (22/61)	
Female	10	60.0 (6/10)	
Age (years)			0.893
≥60	38	39.5 (15/38)	
<60	33	39.4 (13/33)	
Tumor length			0.160
<3cm	7	71.4 (5/7)	
3-5cm	48	37.5 (18/48)	
>5cm	16	31.3 (5/16)	
Tumor location			0.886
Middle	43	37.2 (16/43)	
Lower	28	42.9 (12/28)	
Differentiation			0.886
Well	12	41.7 (5/12)	
Moderately	45	37.8 (17/45)	
Poorly	14	42.9 (6/14)	
pT			0.001
pT1	4	100 (4/4)	
pT2	46	43.5 (20/46)	
pT3	21	19.0 (4/21)	
pN			0.001
-	45	60.0 (27/45)	
+	26	3.8 (1/26)	
pTNM			0.001
pI	4	100 (4/4)	
pII	55	41.8 (23/55)	
pIII	12	8.3 (1/12)	
STAT3			0.074
-	12	66.7 (8/12)	
^+^	59	33.9 (20/59)	
p-STAT3			0.001
-	27	77.8 (21/27)	
+	44	15.9 (7/44)	
VEGF			0.001
-	30	60.0 (18/30)	
+	41	24.2 (10/41)	
CyclinD1			0.002
-	31	61.3 (19/31)	
+	40	22.5 (9/40)	
Bcl-xL			0.114
-	26	50.0 (13/26)	
+	45	33.3 (15/45)	
Chemotherapy,			0.115
-	31	51.6 (16/31)	
+	40	30.0 (12/40)	
Radiotherapy			0.269
-	47	44.7 (21/47)	
+	24	29.2 (7/24)	

*P*, Log-rank test.

**Table 6:** Results of cox regression multivariate 5-year survival analysis.

	B	SE	Wald	P	HR	95.0% CI for HR
Gender	-0.316	0.625	0.255	0.613	0.729	0.214~2.481
Age	-0.364	0.417	0.765	0.382	0.695	0.307~1.572
Tumor length	0.648	0.375	2.987	0.084	1.911	0.917~3.982
Tumor location	-0.117	0.363	0.103	0.748	0.890	0.436~1.814
Differentiation	-0.053	0.308	0.030	0.862	0.948	0.519~1.732
pT	0.459	0.622	0.544	0.461	1.582	0.467~5.357
pN	1.306	0.589	4.906	0.027	3.690	1.162~11.718
pTNM	0.284	0.812	0.122	0.727	1.328	0.271~6.520
STAT3	0.750	0.691	1.179	0.278	2.117	0.547~8.196
p-STAT3	1.158	0.551	4.414	0.036	3.183	1.081~9.372
VEGF	0.761	0.414	3.386	0.066	2.141	0.952~4.818
cyclinD1	0.477	0.513	0.862	0.353	1.611	0.589~4.405
Bcl-xL	0.630	0.445	2.008	0.156	1.878	0.785~4.492
Chemotherapy	-0.650	0.386	2.835	0.092	0.522	0.245~1.113
Radiotherapy	0.090	0.408	0.049	0.825	1.094	0.492~2.432

## Abbreviations

ESCC: Esophageal squamous cell carcinoma
STAT3: Signal transduction and activators of transcription factor 3
VEGF: Vascular endothelial growth factor
